# Enhancing Brain Plasticity to Promote Stroke Recovery

**DOI:** 10.3389/fneur.2020.554089

**Published:** 2020-10-30

**Authors:** Fan Su, Wendong Xu

**Affiliations:** Department of Hand Surgery, Huashan Hospital, Shanghai Medical College, Fudan University, Shanghai, China

**Keywords:** brain restoration, plasticity, stroke, neural bypass, brain remodeling

## Abstract

Stroke disturbs both the structural and functional integrity of the brain. The understanding of stroke pathophysiology has improved greatly in the past several decades. However, effective therapy is still limited, especially for patients who are in the subacute or chronic phase. Multiple novel therapies have been developed to improve clinical outcomes by improving brain plasticity. These approaches either focus on improving brain remodeling and restoration or on constructing a neural bypass to avoid brain injury. This review describes emerging therapies, including modern rehabilitation, brain stimulation, cell therapy, brain-computer interfaces, and peripheral nervous transfer, and highlights treatment-induced plasticity. Key evidence from basic studies on the underlying mechanisms is also briefly discussed. These insights should lead to a deeper understanding of the overall neural circuit changes, the clinical relevance of these changes in stroke, and stroke treatment progress, which will assist in the development of future approaches to enhance brain function after stroke.

## Introduction

Stroke represents the leading cause of long-term disability and causes substantial medical and financial burdens. Even with standard rehabilitation after stroke, the majority of stroke patients are disabled when they enter the chronic phase (1). The lack of effective neurorepair and limitations of functional recovery have led researchers to consider other approaches that improve the scope for recovery by enhancing brain plasticity.

Brain plasticity is defined as the intrinsic ability of the brain to reorganize its function and structure in response to stimuli and injuries. After stroke, the plasticity process is initiated in an attempt to compensate for both the lesion itself and its remote effects. Changed neural activity and connectivity in terms of function and structure could be detected in the perilesional and remote regions and even in the contralateral hemisphere, which were assumed to be the mechanisms underlying spontaneous recovery (2, 3). Generally, increased neural activity and connectivity in the ipsilesional hemisphere were reported as indicators of better functional recovery (4). However, the roles of the recruitment of the contralateral hemisphere seem to be mixed, as both supportive and inhibitory influences of the unaffected hemisphere were detected during recovery. Compensatory or maladaptive processes may contribute to the inconsistency; these processes were attributed to, at least partially, the time since stroke, location and size of the lesion, and other biological factors (e.g., age of the patient) (5, 6).

Various post-stroke interventions have been developed to improve recovery, which either intentionally or not promote the plasticity of the remaining neural circuit. In addition to conventional therapies (i.e., physical, occupational, and speech), novel approaches have shown promising effects in clinical trials. Many of the approaches were designed to enhance plasticity in the ipsilesional hemisphere, in which increased activity/connectivity was related to better functional performance. However, the results obtained thus far are complicated, and even when significant improvements were made, the effect sizes were not satisfactory in most cases.

**Figure 1:**
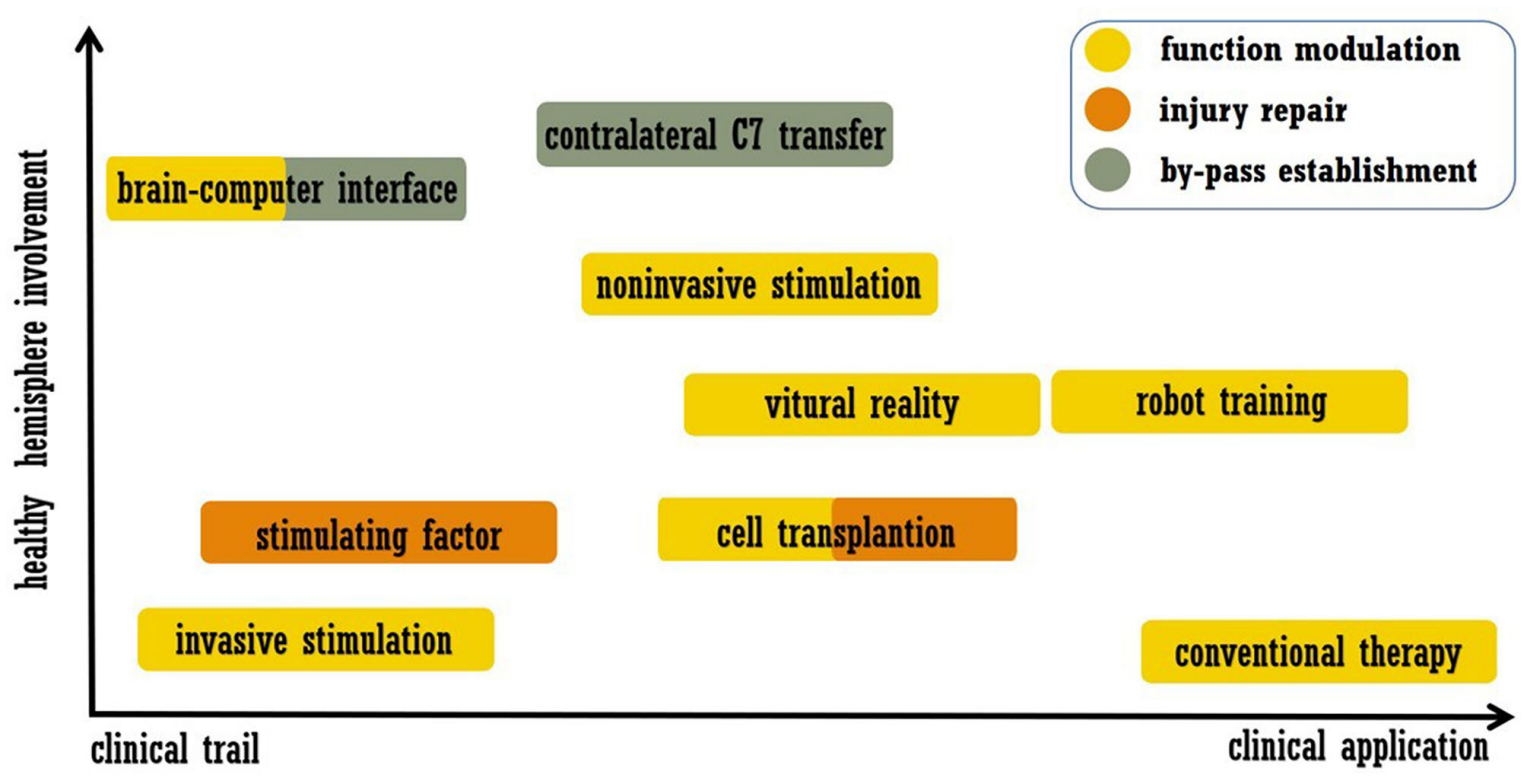
Summary of treatment approaches discussed in this review. The abscissa indicates the readiness of clinical application, and the ordinate indicates the involvement of the healthy hemisphere.

As mentioned above, stroke leads to disturbances across the brain, and interhemispheric interactions have complex influences on functional recovery (6, 7). Accordingly, strategies for recovery may include modulation of the intact hemisphere, and work related to this approach has been performed. In this review, we summarize post-stroke interventions that enhance brain plasticity and functional recovery, placing emphasis on modulation of the intact hemisphere. Acute revascularization and neuroprotection are not included, and approaches that were only utilized in basic researches were also excluded. We searched the PubMed from January 1990 to May 2020. Search key words were combinations of “stroke” and “plasticity or neural plasticity or neuroplasticity or brain remodeling or brain rewire.” The article type was limited to Clinical trial. The final list of references is based on the relevance of the articles to the scope of this review. We did not intend to provide a comprehensive analysis but instead aimed to highlight novel evidence from well-designed trials. In the next section, we discuss the selected examples of promising approaches listed in Figure 1, including modern rehabilitation, brain stimulation, cell therapy, brain-computer interfaces (BCIs), and peripheral nerve transfer. The representative trails that evaluated each intervention were listed in Table 1.

**Table 1:** Representative clinical trials evaluating novel interventions for stroke recovery.

**Trial**	**Stroke phrase**	**No. of subjects**	**Intervention**	**Main outcome**	**Others**
Takahashi et al. (8)	Chronic	13	Robot	Safety, improved arm motor	fMRI: Increased sensorimotor cortex activation after therapy
Ingemanson et al. (9)	Chronic	30	Robot	Safety, improved arm motor	fMRI: Somatosensory system integrity predicts recovery
Pellegrino et al. (10)	Chronic	7	Robot	Improved arm motor	EEG: Modulation of the interhemispheric coherence correlated with recovery
Klamroth-Marganska et al. (11)	Chronic	73	Robot	Safety, improved arm motor	-
Ekman et al. (12)	Chronic	12	VR	Improvement in chronic neglect	fMRI: increased activation in prefrontal and temporal cortex
Xiao et al. (13)	Subacute	8	VR	Improved walking	fMRI: Increased activation in primary sensorimotor cortex correlated with recovery
Saleh et al. (14)	Chronic	19	Robot-Assisted VR	Improved arm motor	fMRI: Brain activity re-lateralized to the ipsilesional side, which correlated with recovery
Orihuela-Espina et al. (15)	Chronic	8	VR	Improved arm motor	fMRI: Increased contralesional activation correlated with recovery
Wang et al. (16)	Subacute	26	VR	Safety and feasibility	fMRI: Increased activation intensity and the laterality index of the contralateral primary sensorimotor cortex
Saposnik et al. (17)	Subacute	141	VR	Safety and improved arm motor	No superiority over recreational activity interventions
Jang et al. (18)	Chronic	5	VR	Improved arm motor	fMRI: Decreased ipsilateral activation and increased contralateral activation
Kim et al. (19)	Chronic	24	VR	Improved balance and walking	-
Stagg et al. (20)	Chronic	24^*^	A-tDCS over affected hemisphere or C-tDCS over unaffected hemisphere	Improved arm motor in both experiments	fMRI: Increased ipsilesional activation correlated with recovery
Chang et al. (21)	Subacute	24	A-tDCS over affected hemisphere	Better lower limb motor (not significant)	MEP: shorter in latency and higher in amplitude
Darkow et al. (22)	Chronic	16	A-tDCS over left M1	No improvement of naming	fMRI: Reduced activity in regions mediating cognitive control; increased language network activity; increased within-network communication
Meinzer et al. (23)	Chronic	26	A-tDCS over left M1	Improved naming and communication	-
Monti et al. (24)	Chronic	8	C-tDCS over left frontotemporal region	Improved naming	A-tDCS failed to improve aphasia
Au-Yeung et al. (25)	Chronic	10	C-tDCS over unaffected M1	Improved hand dexterity	A-tDCS failed to improve motor
Du et al. (26)	Acute	60	LF-TMS over the unaffected hemisphere	Improved arm motor	fMRI: decreased activity in the unaffected cortex
Au-Yeung et al. (27)	Acute, subacute and chronic	49	LF-TMS over the unaffected hemisphere	Improved arm motor	Effectiveness depends on hemispheric dominance
Nowak et al. (28)	Subacute	15	LF-TMS over the unaffected hemisphere	Improved arm motor	fMRI: Decreased overactivity in the contralesional hemisphere; overactivity of the contralesional hemisphere predicted recovery
Grefkes et al. (29)	Subacute	11	LF-TMS over the unaffected hemisphere	Improved arm motor	fMRI: Reduction of interhemisphere inhibition correlated with motor recovery
Kondziolka et al. (30)	Chronic	18	Intracranial delivery of NT2	Safety, feasibility and improved arm motor	-
Prasad et al. (31)	Subacute	120	Intravenous delivery of BMSCs	Safety and feasibility; no improvement in stroke outcome	-
Friedrich et al. (32)	Acute	20	Intraarterial delivery of BMSCs	Safety and feasibility	Some patients showed good outcome
Savitz et al. (33)	Subacute	100	Intracarotid delivery of ALD-401	Safety and feasibility; no improvement of stroke outcome	Smaller lesions in treatment group (no significant)
Steinberg et al. (34)	Chronic	18	Intracerebral implantation of SB623	Safety; improved stroke outcome	-
Muir et al. (35)	Subacute and chronic	23	intracerebral implantation of CTX0E03	Safety, feasibility, improved arm motor	-
Sharma et al. (36)	Chronic	24	Intrathecal delivery of BMSCs	Safety; improved arm motor and balance	-
Fang et al. (37)	Acute	18	Intravenous delivery of EPCs	Safety and feasibility	No significant improvement of stroke outcome
Ramos-Murguialday et al. (38)	Chronic	30	BCI	Improved arm motor	fMRI: No significant difference
Wu et al. (39)	Subacute	25	BCI	Improved arm motor	fMRI: Increased neural activity across the whole brain; inter-hemispheric connectivity correlated with recovery
Pichiorri et al. (40)	Subacute	28	BCI	Improved arm motor	EEG: Ipsilesional intrahemispheric connectivity correlated with recovery
Carino-Escobar et al. (41)	Subacute and chronic	9	BCI	Improved arm motor	EEG: Longitudinal Trends ERD/ERS correlated with recovery
Hua et al. (42)	Chronic	12	CC7	Safety and improved arm motor	-
Zheng et al. (43)	Chronic	36	CC7	Safety and improved arm motor	TMS and fMRI: Connectivity between the ipsilateral hemisphere and paralyzed arm; establishment of functional regions in ipsilateral hemisphere to control paralyzed arm
Qiu et al. (44)	Chronic	2	Contralateral lumbar-to-sacral nerve rerouting	Safety and improved ambulatory status	-

* *The trial consisted of two experiments. Experiment 1 enrolled 13 participants and experiment 2 enrolled 11 participants. Seven participants took part in both experiments, which were performed more than 1 year apart*.

*VR, virtual reality; A-tDCS, anodal transcranial direct current stimulation; C-tDCS, cathodal transcranial direct current stimulation; LF-TMS, low-frequency transcranial magnetic stimulation; BMSCs, bone marrow mononuclear stem cells; SB623, modified bone marrow-derived mesenchymal stem cells; CTX0E03, human neural stem cell line; ALD-401, enriched population of aldehyde dehydrogenase-bright stem cells; EPCs, endothelial progenitor cells; BCI, brain-computer interfaces; CC7, contralateral seventh cervical nerve transfer; ERD/ERS, event-related desynchronization or synchronization*.

## Main Text

### Modern Rehabilitation

Conventional rehabilitation approaches, such as physical therapy for motor disability and speech and language therapy for aphasia, have been widely practiced. Meta-analyses and recent large-scale randomized controlled clinical trials suggested that therapy is beneficial if it has a high intensity, involves a high dose or occurs over a long period of time (45, 46). Therapy-induced plasticity was suggested to mediate recovery. Generally, increased structural connectivity in both hemispheres correlated with better performance, and functional reactivation on the affected side promoted recovery (2, 47, 48). Two forms of rehabilitation are expected to exceed the efficacy of conventional approaches, i.e., constraint-induced movement therapy (CIMT) and mirror therapy/action observation therapy (49–51). In CIMT, the constraint of the unaffected limb relieved the learned disuse and modified the amendment of maladaptive plasticity across the brain (52). Mirror therapy improved post-stroke recovery on the basis of enhancing the mirror neuron system, which refers to the neurons that are involved in the performance of the observed actions (53). In addition to physiotherapy, advances in modern technology have enriched the means of promoting post-stroke recovery. The combinations of physiotherapy and new technologies have been extensively tested in clinical trials. For example, telerehabilitation is an emerging field owing to the advancement of telehealth, which enables patients to receive home-based, high-dose supervised practice. The field is still emerging, and meta-analysis has suggested that telerehabilitation may be a promising intervention aimed at improving multiple functional deficits after stroke; however, more studies are needed to draw definitive conclusions (54, 55).

#### Robot-Assisted Training

Electromechanical and robot-assisted training are of particular interest, and studies have been performed to explore their efficiency. According to a recent Cochrane review, high-quality evidence supports that robot-assisted arm training improves activities of daily living, arm function, and muscle strength in stroke survivors (56). For lower limbs, patients receiving electromechanical-assisted gait training combined with physiotherapy were more likely to walk independently (57). The main reason for the robot-induced improvements was speculated to be the motivation due to the feedback of the device, the novelty of a robotic device, or both. Although concern remains as to whether robot-assisted therapy is more beneficial than traditional interventions at the same intensity, specific brain plasticity was found during robot-assisted therapy. For patients with stroke, increased ipsilateral sensorimotor cortex activation was detected when performing the practiced task delivered by a robot (8), and ipsilateral somatosensory integration strongly modified hand function gains after robot-assisted training (9). Furthermore, interhemispheric plasticity was also shown to be involved in the recovery process. For instance, a relationship was reported between motor recovery and the normalization of the interhemispheric connectivity between bilateral primary somatosensory areas (10). Further, in stroke models, the combination of robot-assisted training and inactivation of the unaffected hemisphere contributed to significantly improved motor function compared to that achieved with robot-assisted training alone, and functional recovery was accompanied by significantly reduced interhemispheric inhibition (IHI) (58). In brief, robot-assisted therapy appears to be effective for patients with stroke, and the modification of plasticity in both hemispheres seems to mediate functional recovery. Nevertheless, it should be noted that the absolute improvements of robotic therapy were small in some investigations, which limits its clinical promotion (11).

#### Virtual Reality

In addition to robotic therapy, virtual reality (VR) technology has also been widely tested in post-stroke therapy. Numerous clinical studies have been conducted in the field, and the latest meta-analysis suggests that when combined with traditional therapy, VR technology can further improve the prognosis of patients, especially in regards to the dimensions of their upper limb motor function and daily living ability (59). Favorable results were also reported in improving post-stroke balance, gait and neglect (12, 60). At the mechanistic level, VR-induced neuroplasticity has also been confirmed. Similarly, increased recruitment of the ipsilesional hemisphere may mediate functional recovery. For instance, enhanced activation in the ipsilesional sensorimotor cortex correlates with motor improvement (13), and increases in frontal neuronal activity may reflect the plasticity processes underlying positive rehabilitation effects for chronic neglect (12, 61). VR treatment can also induce plasticity in the contralateral hemisphere and cerebellum. On the one hand, functional relateralization to the ipsilesional side was reported to support recovery (14, 62), while on the other hand, improved overall brain activity (including that in the contralateral cortex and cerebellum) was also suggested to drive recovery (15, 16). Although specific neural patterns of reorganization were highlighted by the above trials, there was insufficient evidence to reach definitive conclusions about VR-induced plasticity. Additional studies should address specific questions about the type, timing, frequency, and duration of modern tech-assisted therapy. A deeper understanding of the treatment-related mechanisms and large, definitive and pragmatic phase III trials should be encouraged. Individualized treatment and the use of multiple techniques in combination (such as VR and BCI) (63) are also worth investigating.

Compared with conventional rehabilitation, VR and robot-assisted therapy may advantageously increase the frequency and motivation of rehabilitation training and independent exercise (64, 65). Furthermore, it also enables the participants to practice everyday activities that are not available in the hospital (59). However, a decline in the participant's medical condition (e.g., treatment-unrelated infection), private reasons (e.g., difficulty traveling) and absence of a physiotherapist may limit access to therapy. Although no serious side effects related to treatment have been reported thus far, participants may experience pain, dizziness, headache and high tension after therapy (56, 57, 59).

### Brain Stimulation

Brain stimulation represents a promising area of research because it allows the excitability of the target area to be manipulated directly; pertinently, techniques including transcranial magnetic stimulation (TMS), transcranial direct current stimulation (tDCS), cortical microelectrode stimulation and deep brain stimulation (DBS) have been utilized in this field. Compared to non-invasive stimulation approaches, invasive approaches can deliver stimulation for longer periods at more precise locations. Pilot clinical studies have demonstrated safety and feasibility for implanted cortical electrical stimulation in the ipsilesional M1 region, and improved arm function has also been suggested (66). Evidence has suggested that DBS can help manage various post-stroke maladaptive disorders, especially reducing pain (67).

#### TMS/tDCS in Stroke Treatment

Non-invasive stimulations have been more widely used, and the stimulation parameters determine whether the stimulation increases or decreases brain activity. Generally, anodal tDCS (A-tDCS) has been shown to enhance cortical activity, whereas cathodal tDCS (C-tDCS) usually has the opposite effect. Depending on the frequency, repetitive TMS (rTMS) can reduce [low frequency (LF): ~1 Hz] or increase [high frequency (HF): 5~20 Hz] corticospinal excitability. Both tDCS and rTMS have been used to treat various post-stroke deficits, particularly motor impairment and aphasia. Meta-analyses may imply the effectiveness of non-invasive stimulations, and recent trials with randomized, blind and controlled designs have encouraged further investigation in this field (68–70). For example, Fridriksson et al. (71) reported that activating the left hemisphere by A-tDCS in chronic stroke patients with aphasia led to a relative 70% increase in correct naming compared to that in the sham tDCS group; this effect lasted for 24 weeks after treatment. For acute stroke patients with motor impairment, enhancing cortical activity via HF-TMS and A-tDCS resulted in improved motor function, and stimulation-induced increased neural activity correlated with recovery (21, 72).

**Figure 2:**
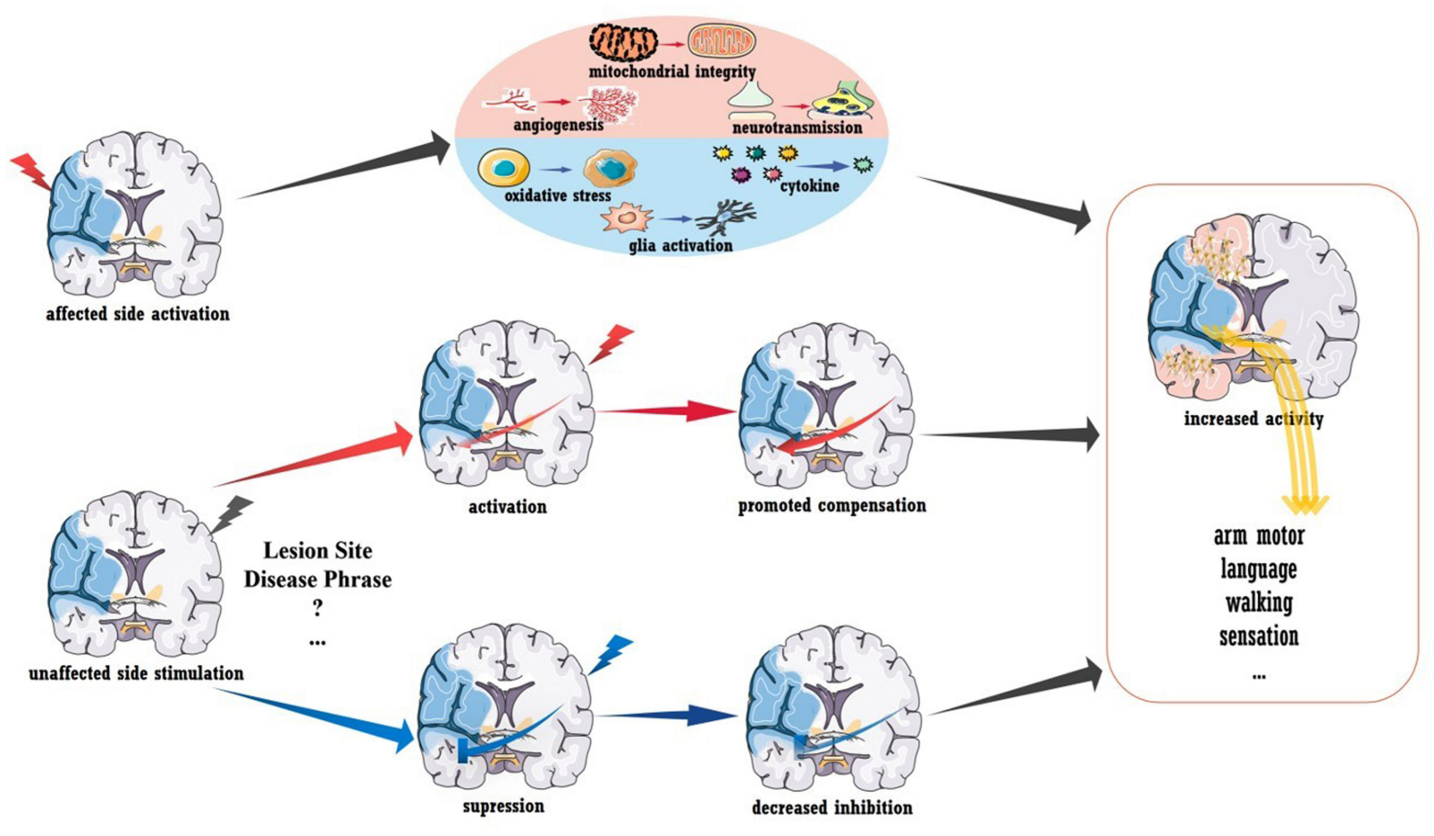
Brain stimulation to promote stroke recovery. Activation of the injured hemisphere can improve prognosis, and the underlying mechanisms may include the promotion of angiogenesis, mitochondrial integrity and neurotransmission and the inhibition of glial activation, pro-inflammatory mediator secretion and oxidative stress. Healthy hemisphere stimulation could have various influences on stroke recovery. Increased interhemispheric compensation (induced by activated stimulation) or reduced interhemispheric suppression (induced by inhibitory stimulation) may mediate clinical recovery.

Despite the exciting results described above, the use of TMS/tDCS as a clinical treatment after stroke seems premature (73). As shown in Figure 2, A-tDCS and HF-TMS over the injured hemisphere paired with rehabilitation is theoretically assumed to promote post-stroke recovery. However, no definite conclusion has been obtained in regards to aphasia and upper/lower extremity function (68, 74), as more evidence from randomized trials is required. Similarly, the outcomes of stimulating the unaffected hemisphere were also mixed. According to the IHI model, stroke disrupts the interhemispheric balance, and the unaffected hemisphere becomes overactive, which further inhibits the activity of the injured hemisphere (75). Thus, theoretically, suppressing the unaffected hemisphere could also be beneficial for stroke recovery, and evidence supports this concept. For stroke patients in the acute phase, LF-TMS over the unaffected hemisphere in the M1 area led to improved arm function compared to that observed after sham stimulation; this improvement seemed to be mediated by the decreased neural activity in the M1 area of the unaffected hemisphere as measured by fMRI (26). Similar results were also reported in independent participants within 6 months of stroke onset (27, 28). However, inconsistent data were also reported (76), and a reason for the contradiction may be that the inhibitory protocols were effective only for patients with abnormal IHI. As shown by Grefkes et al. rTMS-induced inhibition of the contralesional M1 area showed the greatest therapeutic effects in patients who showed the strongest reduction in IHI as measured by fMRI (29).

The advantage of TMS and tDCS is the non-invasive, precise regulation of excitability within specific brain regions. However, some concerns remain regarding clinical applications that need to be further explored, including the stimulation target, therapeutic time point, and stimulation frequency and parameters (73). Regarding safety, both interventions have been suggested to be safe and well-tolerated. Common adverse effects include dizziness, headache, transient aching and burning sensations. Skin reactions at the electrode contact site are also reported. Serious adverse events, such as epileptic seizures, have rarely been reported in related trials (68, 77).

#### TMS/tDCS-Induced Plasticity

To conclude, more work is needed to demonstrate the efficiency of brain stimulation in post-stroke recovery. Revealing the mechanism underlying plasticity will certainly be advantageous. In addition to the stimulation-induced functional remodeling mentioned above, increased structural connectivity was also detected in a recent preliminary diffusion tensor imaging (DTI) study. As reported, acute patients receiving rTMS showed increased fractional anisotropy (FA) in the contralesional corticospinal tract and bilateral cerebellum (78). In rodent models with acute stroke, stimulation-induced recovery was related to the inhibition of pro-inflammatory cytokines, oxidative stress, and glial activation as well as to the promotion of mitochondrial integrity, angiogenesis, and neurotransmission (shown in Figure 2) (79, 80). The modulation of long-term potentiation and long-term depression has been demonstrated *in vitro*, and the brain-derived neurotrophic factor (BDNF)-mediated signaling pathway is involved in this process (81, 82). More importantly, stimulation-induced plasticity should be viewed at the “whole-brain” level rather than at areas of local activation or inhibition. A pilot clinical study reported that activating the premotor cortex and supplementary motor helped the recruitment of the contralesional hemisphere (83). Furthermore, contralesional M1 inhibition could also improve coupling between the premotor cortex and M1 area in the ipsilesional brain (29), and stimulating the left M1 area was reported to enhance language function in chronic stroke patients with aphasia (23). These interactions between different functional areas (i.e., motor-language interaction) and the interhemispheric modulations are believed to mediate functional recovery. Understanding post-stroke and treatment-related plasticity from a holistic and dynamic perspective is key to promoting brain stimulation therapy, and the combination of multiple techniques, such as fMRI, may be an effective approach.

### Cell Therapy

Cell therapy is emerging as a viable neurorestorative therapy for stroke, and the number of investigations in this field have surged in recent decades. Stem cells are self-perpetuating and have the ability to transform into multiple cell types. Theoretically, both endogenous and exogenous therapeutic strategies are capable of promoting post-stroke plasticity. The former refers to increasing mobilization, longevity and autologous stem cell production, and the therapeutic targets have focused on neurotrophic and growth factors, inflammatory circumstances, and chemokine receptors (84). Currently, most research in this research has been limited to preclinical studies except for that regarding growth factors. However, according to a recent meta-analysis, there are significant safety concerns regarding the stimulating factors (including erythropoietin, granulocyte colony-stimulating factor and analogs) for patients with acute or subacute stroke (85). Therefore, whether the above treatment can be used in the clinic needs further clarification.

#### Exogenous Cell Transplantation in Stroke Treatment

In contrast, exogenous cell therapy provides the brain with donor cells, the sources of which are generally divided into three types: immortalized cell lines, neural progenitor or stem cells, and bone marrow-derived progenitor or stem cells (86). Through various administration routes, cells have been assessed in human studies for their ability to improve post-stroke disability. Previous reviews have summarized the evidence to support the safety, feasibility and effectiveness of various cell types, and recent publications have added to our knowledge on this topic (86, 87). The NT2N cell line, derived from teratocarcinoma cells, was first used in clinical trials. By intracerebral administration of the cells into peri-infarct or peri-hemorrhagic regions, safety and functional improvements for patients with chronic stroke were suggested in phase I/II trials (30). Regarding bone marrow mononuclear stem cells (BMSCs), intravenous infusion was tested in a multicenter, randomized trial that included 120 patients with subacute ischemic stroke. Safety was suggested in this trial, but no beneficial effect on stroke outcome was observed (31). Alternative administration (intra-arterial) of BMSCs might be effective, as suggested by a pilot study (88), and the phase II trial is currently ongoing (registered no. NCT02178657 at ClinicalTrials.gov). Furthermore, a recent report showed that intra-arterial BMSC transplantation may modulate circulating miRNA-34a levels; this modulation was related to precursor cell migration and infarct volumes in stroke patients (89). For ALD-401 cells, another type of bone marrow-derived stem cell, intracarotid infusion may be promising for subacute ischemic stroke patients. A recent phase II trial suggested that intracarotid infusion of ALD-401 did not lead to adverse events, and the mean difference in recovery favored the treatment group; however, this difference failed to reach statistical significance (33). The large standard deviation in participants may have contributed to the negative result, and the fact that the trial was terminated when it reached the safety endpoint was also related to the results (90). Phase II trials for bone marrow-derived mesenchymal stem cells (SB623) and human neural stem cells (CTX0E03), which were intravenously and intracellularly implanted, respectively, were completed, and the encouraging results motivated further trials for both cell types (34, 35). Intrathecal injection of bone marrow-derived stem cells was also shown to be safe, and improved functional recovery accompanied by increased brain metabolic activity was suggested by a non-randomized, single-arm trial (36). Further randomized, controlled, blinded trials are in process (registered no. ChiCTR-INR-16008908 at ChiCTR).

In addition to the replacement of dead cells in the infarct area by transplanted cells, the advantages of cell therapy may also be related to the benign regulation of the whole brain microenvironment and plasticity (86, 87) (details in the next section). Regarding safety, despite inconclusiveness between the cell therapy and control groups (91), some potential serious adverse effects should be noted, including neoplastic transformation, worsening of neurological deficit, epilepsy, and even death (91, 92). Moreover, transplantation-related side effects may also include infection, hemorrhage, nausea and vomiting, depression, fatigue and increased blood glucose and CRP levels (93, 94).

#### Cell Therapy-Induced Plasticity

**Figure 3:**
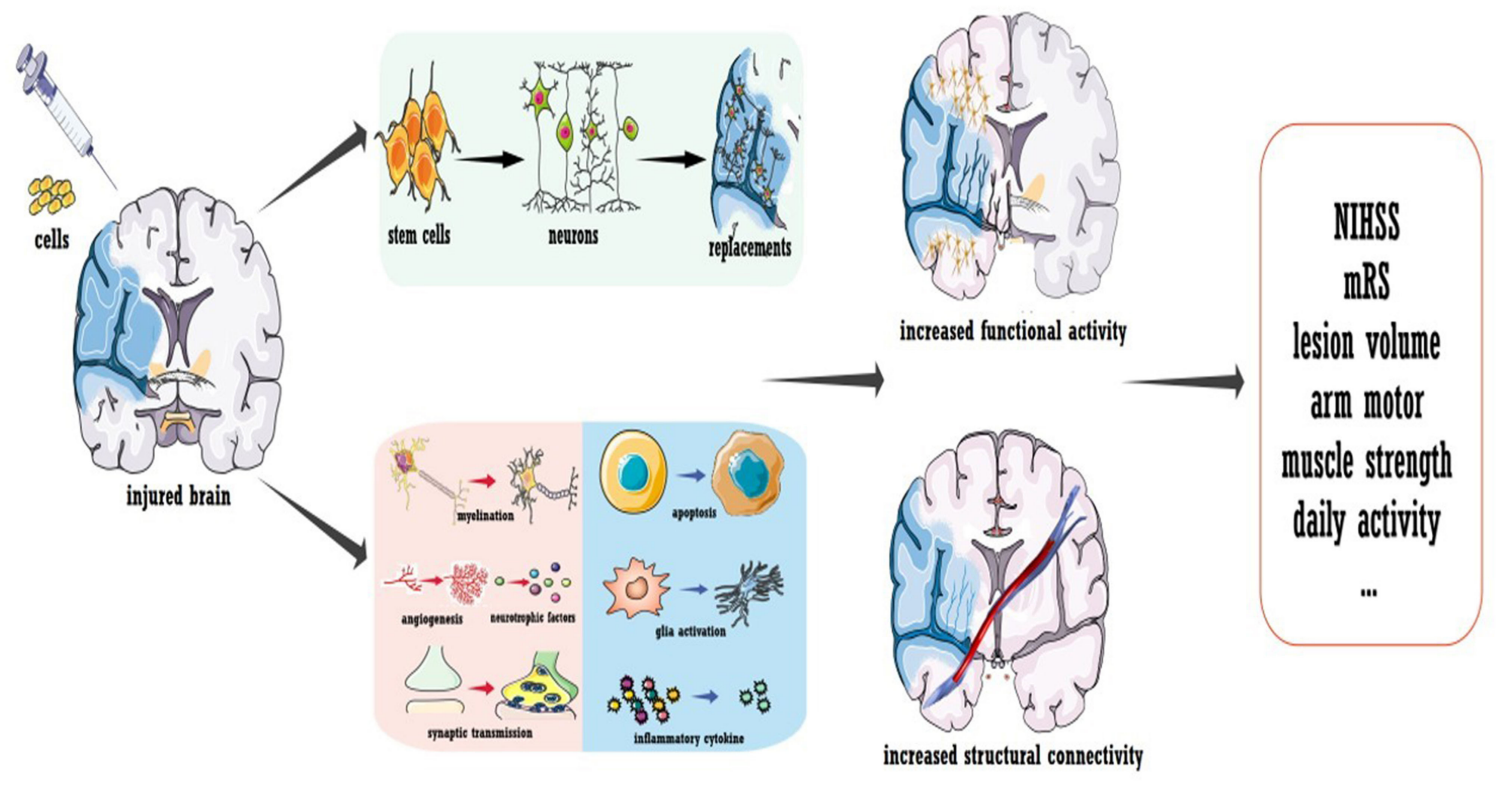
Cell transplantation to promote stroke recovery. Cell therapy was shown to induce the replacement of dead neurons in the infarcted area; more importantly, it ameliorated the microenvironment of the whole brain to promote functional modulation. Treatment not only enhances the neural activity of the injured hemisphere but also improves the structural connection of the whole brain.

To conclude, a few questions remain regarding cell therapy for stroke, but the promising results obtained thus far strongly inspire further trials. Several concerns should be carefully addressed in future phase IIb/III investigations, especially regarding the sham procedure and blindness. As illustrated in Figure 3, the mechanism underlying cell therapy was originally assumed to be the replacement of injured brain cells by transplanted cells. However, not all evidence supports this concept, especially for cells with a reduced capacity for neural differentiation. It was suggested that ~1/3 of locally injected cells migrate to the focal infarct area (95, 96), while for systematic delivery, <10% exogenous cells arrive at the lesion area (97). Additionally, only a small proportion of migrated cells were shown to differentiate into mature neurons, and the stem cell source dictated the proportion (95, 96, 98). The new neurons were estimated to replace only ~0.2% of the dead neurons due to stroke (99). Furthermore, behavioral improvements were not always related to the number of cells that integrated into the circuit and the timing of synapse formation (100). More importantly, functional recovery independent of integration has also been reported (101). Thus, even though transplanted cells could replace the injured circuit, cell therapy also promoted recovery via other mechanisms. As discussed in other reviews (86, 87), the majority of functional recovery achieved with stem cells was attributed to the enhancement of axonal myelination and synaptic transmission, promotion of neurogenesis and angiogenesis, increased secretion of neurotrophic/growth factors, immunomodulation, reduced apoptosis, maintenance of the blood-brain barrier, and reconstruction of white matter (listed in Figure 3). Notably, cell therapy-induced plasticity occurred across the brain. Regarding inflammation and neurotrophic/growth factors, favorable microenvironments were found in both hemispheres and even in the peripheral circulation (86, 102, 103). Landmark evidence was acquired regarding axonal plasticity. For both neural stem cells and bone marrow-derived cells, cell-grafted rats demonstrated increased widespread axonal rewiring from the contralesional side, with transcallosal and corticospinal axonal sprouting correlating with functional recovery (104, 105).

These mechanisms need to be further elucidated. The gap between stroke patients and animal models should be stressed, and it seems that most basic studies focused on acute or subacute stroke. Regarding the different pathophysiological events between acute and chronic stroke, these results may play a limited role in promoting cell therapy for chronic stroke, which has great clinical importance. Furthermore, optimizing the delivery methods and *in vivo* tracking of implanted cells are of great significance.

### Brain-Computer Interfaces

#### Clinical Application of BCI

In contrast to the treatments mentioned above, BCIs constitute a novel idea for functional recovery after stroke, as they avoid the injured area, build a neural bypass based on a healthy brain, and promote dominance in some brain regions. Based on its methodology of measuring brain activity, BCI use can be invasive or non-invasive, and electrical, magnetic or metabolic neuron activity can be recorded. The detected signals are further amplified, filtered, decoded and translated into signals for controlling the external devices or directly stimulating the muscles or the brain (106). The closed-loop system can replace, restore or enhance natural neural output, thereby improving lost function due to brain injury. Although large-scale randomized trials are currently scarce, promising results have been obtained in this field. According to a recent meta-analysis, BCI training was associated with a larger improvement of upper limb motor function (107), which was supported by several other investigations (38, 39). Theoretically, BCI training engages learning and neural adaption processes, and brain remodeling has been detected by posttreatment MRI or electrophysiological tests in stroke patients (108). Overall, increased activity and connectivity in the ipsilesional brain were detected and correlated with behavioral improvements. As suggested by Pichiorri et al. BCIs enhance electroencephalography sensorimotor power spectra and motor improvements associated with increases in ipsilesional intrahemispheric connectivity (40). Furthermore, plasticity may occur in broader brain regions. As determined by both electroencephalography and MRI, increased interhemispheric recruitment correlated with upper limb motor recovery (41). These results may reinforce the hypothesis that broader activation during movement tasks is a form of compensation observed in patients with stroke.

Since BCI was first used in stroke rehabilitation in 2009 (109), evidence supports its safety and effectiveness (106). BCI can be well-integrated with other modern rehabilitation approaches, such as robots, tDCS and motor imagery, providing more feasibility for clinical application (110). However, it should be noted that only a small number of studies explored the long-term effect of BCI in stroke rehabilitation, and this concept needs further validation (110). Moreover, BCI training requires a high degree of concentration and self-regulation; thus, patients with post-stroke emotional or cognitive impairments may not be able to cooperate. To date, no serious BCI-related adverse events have been reported, and common treatment-related side effects include transient nausea, fatigue, and headaches (110, 111).

#### Basic Research Reveals BCI-Induced Plasticity

Clinical evidence suggests that BCI allowed increased coordination between the multisensory and motor-related cortex and the extrapyramidal system. By inducing neuroplasticity and restoring lost function, BCI represents a feasible option for post-stroke rehabilitation. Exploring BCI-induced plasticity can not only promote post-stroke rehabilitation but also expand our understanding of the mechanisms underlying motor learning. Basic experiments in non-human primates have shed light on this field. As summarized by Shanechi, there may be two neural mechanisms for BCI (108). On the one hand, training directly changed the activity of individual output neurons based on neurofeedback to achieve behavioral goals. Compared with nearby neurons, output neurons exhibited different changes in activity after BCI training. Changes in corticostriatal interactions that are specific to the output neurons were also involved in BCI training (112). On the other hand, training explored an existing pattern of neural activity related to natural movements and then reassociated them, thus serving as a reorganization strategy. This concept was supported by the finding that animals learned to control single-neuron activity by preferentially exploring and exploiting the natural movement repertoire (113). Furthermore, in the population-level changes of neural activity, animals relied on a fixed repertoire of activity patterns and associated those patterns with various movements after learning (114). There was evidence that the seemingly inconsistent processes cooccurred on different time scales in BCI-mediated motor training (108).

The substantial amount of work in preliminary trials and animal models has demonstrated the potential of BCIs to restore upper limb function after stroke. To validate the observed efficiency, more large-scale, randomized, and controlled trials with long-term follow-ups in stroke patients are urgently needed. In addition, BCIs may also play a role in restoring multiple post-stroke functions, such as walking, communication and mood (106, 108, 115). Furthermore, the combination of BCIs and other technologies, such as paired associative stimulation and VR, may represent a feasible approach to optimize BCI treatment (63). The above investigations are still in the initial stage, and only studies in large patient groups can lead to definite conclusions about the value of BCI in specific patient subsets.

### Peripheral Nerve Transfer

#### Contralateral Seventh Cervical Nerve Transfer in the Treatment of Spastic Arm Paralysis

**Figure 4:**
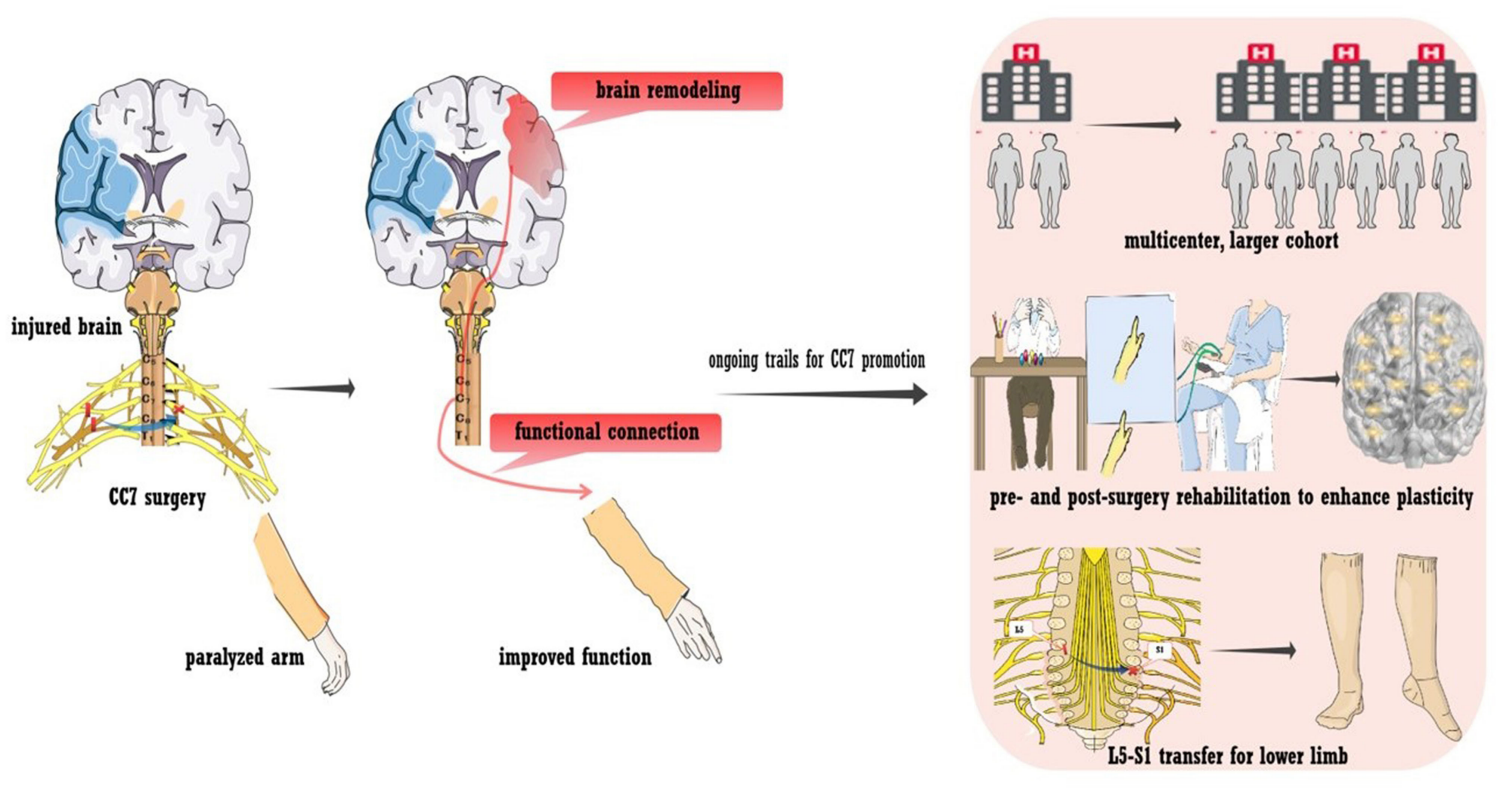
Contralateral seventh cervical nerve transfer (CC7) to promote stroke recovery. A neural bypass was constructed via CC7 surgery to functionally connect the paralyzed hand and healthy hemisphere. Various trials are ongoing regarding CC7 surgery, including a large-sample multicenter trial, L5-S1 transfer to the lower limb, and pre- and post-surgery rehabilitation to facilitate plasticity.

An alternative way to bypass a lesion is contralateral seventh cervical nerve transfer (CC7), a surgical approach developed by our team (43). The principle of the surgery is briefly outlined in Figure 4. During the operation, the seventh cervical nerve (C7) from the non-paralyzed side is transferred to the paralyzed side, which enables the development of functional connections between the contralesional hemisphere and the paralyzed arm. This treatment approach has been used for injury to the brachial plexus since the 1980s (116). In addition to the improvement of upper arm function, we also detected post-surgery changes in brain activity (117–119). This phenomenon indicated that rewiring the peripheral nerve connection led to brain plasticity, which supported us in employing surgery to treat spastic arm paralysis (42). As detected by our recent trial enrolling participants with chronic brain injury (25% were stroke patients), CC7 surgery in combination with conventional rehabilitation was associated with a mean increase in the Fugl-Meyer score of 17.7 points (43). The improvement was significantly higher than that of the control group, who received rehabilitation alone, and greatly exceeded the minimal clinically important differences (MCID) for the upper extremity of 5.25 points in chronic stroke cases (120). The surgery induced brain plasticity, mediating functional recovery. The MRI scan showed that at postoperative month 8, voluntary extension of the paralyzed wrist generated neural activation in the contralesional hemisphere. Activation increased in amplitude at postoperative months 10 and 12, while the activation of the ipsilesional hemisphere decreased at postoperative month 12 compared with that at baseline. Furthermore, stimulating the contralesional hemisphere via TMS induced motor-evoked potential in the paralyzed arm at postoperative months 10 and 12. This evidence demonstrates the functional connections between the contralesional hemisphere and the paralyzed arm (indicated in Figure 4) (43).

To date, no serious adverse events related to CC7 have occurred. In addition to general surgically related adverse reactions, such as nausea and vomiting, pain in the limb or shoulder, foreign-body sensation while swallowing and fatigue are major adverse events after CC7. Additionally, numbness in the hand, decreased power of elbow/wrist extension and attenuated sensory function occurred on the side of the donor nerve due to neurotomy. All these deficits were almost completely relieved at post-surgery month 3 (43). In the future, we must pay attention to potential risks, including long-term decreases in sensory or motor function, and long-term follow-up is needed to confirm the safety of CC7 surgery.

#### CC7 in Optimization

Innovative work allows the control of the contralesional hemisphere over the paralyzed arm. In addition to promoting recovery, CC7 surgery also provides opportunities for insights into basic neuroanatomy and neurophysiology. Figure 4 also shows ongoing trials to promote the treatment approach. First, multicenter trials enrolling larger cohorts with longer follow-ups are in progress to determine whether CC7 results in safe, consistent, and long-term functional improvements. The large sample size will also enable analysis of multidimensional clinical characteristics, exploration of factors that may affect the efficacy of surgery, and provision of a basis for individualized treatment. Second, peripheral nerve transfer has been utilized in treating lower limb dysfunction due to brain injury. Preliminary studies have suggested the feasibility and effectiveness, which needs to be further verified (44). Third, owing to the unique postoperative changes, these traditional rehabilitation approaches may lack a feasible template. The rehabilitation program that targets postoperative dynamic plasticity was assumed to improve efficiency (121). Finally, we hypothesized that intensive preoperative rehabilitation improves brain activity, fully mobilizes brain reserve capacity, and provides a better foundation for postoperative brain remodeling. Thus, a trial testing the effect of preoperative intensive rehabilitation for CC7 is in progress. Last, we are also investigating the mechanisms that underlie brain plasticity in animal models.

Additional evidence will help to confirm the safety and efficiency of CC7, suggest mechanisms underlying brain plasticity, and establish a better CC7 treatment scheme.

## Conclusions

Plasticity is a natural property of the human brain, and its lifelong capacity enables us a much longer therapeutic window for post-stroke neural restoration than previously assumed. As summarized in previous reviews (84, 122, 123), the modulation of plasticity to promote post-stroke recovery has increasingly advanced in recent decades. This current review updates the previous ones by discussing the results from the latest trials. Key evidence from basic studies was also highlighted to provide a more comprehensive overview. Furthermore, stroke is a complicated disease affecting various brain regions, resulting in disruption of entire brain networks and widespread dysfunctions. A study targeting only certain molecular pathways or areas surrounding the injury may have limited significance. Some innovative ideas, such as BCI and CC7, that induce and employ plasticity in the healthy hemisphere may be of significance, and the promotion and optimization of these novel approaches may contribute to stroke therapy.

Emerging evidence has renewed our knowledge of post-stroke therapy. As discussed in the review, approaches that support injured neural circuits or rewire neural pathways have been developed. Exciting improvements in clinical function were achieved, accompanied by the successful induction of plasticity across the whole brain. Despite the exciting achievements, further investigations are strongly encouraged. The clinical trialists are still looking to test more novel interventions, as the increased understanding of stroke recovery and development of techniques to measure and enhance brain plasticity will continue to promote the interventions. Moreover, the inconsistent results have been constantly reported in trials that evaluating each intervention for stroke recovery. The heterogeneity of participants enrolled and the parameters of treatment may be related to the inconsistency, which should be illustrated in future trial. For instance, the timing for the intervention should be considered. The traditional concept suggests that the degree for improving plasticity is larger in earlier stages than in later stages. Accordingly, many trials tended to enroll patients in the early phase (<6 months) after stroke onset. However, the stressful microenvironment in the early post-stroke stage may inhibit treatment-induced plasticity. Indeed, much evidence has challenged “the earlier the better” principle (123). Additionally, priority should also be given to the individualized treatment, which require a deeper understanding of the pathophysiological process and better measurement of brain activity for participants. The trialists may consider the application of genomics, proteomics, neuroimaging and electrophysiology in future trial. Meanwhile, advancements in basic research will substantially contribute to post-stroke therapy. On one hand, novel therapies are beginning to emerge from the basic research. For example, optogenetics is a revolutionary neuroscience tool that uses bioengineered light-sensitive proteins to selectively activate or inhibit specific cell types and neural circuits, and the optogenetic stimulation to enhance stroke recovery has been widely utilized in animal models (124). There are vast gaps between clinical use and basic research, and the therapy found to be effective in animals may not be efficient in human patients. More work, such as the development of animal model that more accurately reflect the human brain, are needed to overcome the challenge for translating animal study to clinical practice. For example, even with the advanced human brain mapping techniques (such as fMRI and TMS), the microstructural changes at the level of axons and synapses after therapy are still not available. Information from animal models help to provide a comprehensive picture of therapy-induced plasticity, which guides the clinic application of novel intervention. In a word, advancements in both clinical and basic research are required to the development of stroke therapy and the reveal of mechanisms underlying therapy-induced plasticity.

To conclude, the adult brain has strong plasticity potential, which can be exploited by therapeutic approaches in patients with stroke. Various approaches aimed at stimulating or inducing beneficial plasticity have been indicated to be effective. Further progress in this field requires a deeper understanding of the neural circuit changes and their functional implications at the whole-brain level as well as complete elucidation of treatment-induced plasticity.

## Author Contributions

WX contributed to the concept of this review and revised the manuscript. FS carried out the literature review and drafted the manuscript. Both authors read and approved the final manuscript.

## Conflict of Interest

The authors declare that the research was conducted in the absence of any commercial or financial relationships that could be construed as a potential conflict of interest.
